# Stress and Burnout in Health-Care Workers after the 2009 L’Aquila Earthquake: A Cross-Sectional Observational Study

**DOI:** 10.3389/fpsyt.2017.00098

**Published:** 2017-06-12

**Authors:** Antonella Mattei, Fabiana Fiasca, Mariachiara Mazzei, Stefano Necozione, Valeria Bianchini

**Affiliations:** ^1^Department of Life, Health and Environmental Sciences, University of L’Aquila, L’Aquila, Italy; ^2^Department of Mental Health—Residences for the Execution of Security Measures, Subiaco, Rome, Italy

**Keywords:** burnout, stress, work, health care, prevalence, association, risk factor, earthquake

## Abstract

Burnout is a work-related mental health impairment, which is now recognized as a real problem in the context of the helping professions due to its adverse health outcomes on efficiency. To our knowledge, the literature on the postdisaster scenario in Italy is limited by a focus on mental health professionals rather than other health-care workers. Our cross-sectional study aims to evaluate the prevalence of burnout and psychopathological distress in different categories of health-care workers, i.e., physicians, nurses, and health-care assistants, working in different departments of L’Aquila St. Salvatore General Hospital 6 years after the 2009 earthquake in order to prevent and reduce work-related burnout. With a two-stage cluster sampling, a total of 8 departments out of a total of 28 departments were selected and the total sample included 300 health-care workers. All the participants completed the following self-reporting questionnaires: a sociodemographic data form, a Maslach Burnout Inventory and a General Health Questionnaire 12 Items (GHQ-12). Statistically significant differences emerged between the total scores of the GHQ-12: *post hoc* analysis showed that the total average scores of the GHQ-12 were significantly higher in doctors than in health-care assistants. A high prevalence of burnout among doctors (25.97%) emerged. Using multivariate analysis, we identified a hostile relationship with colleagues, direct exposure to the L’Aquila earthquake and moderate to high levels of distress as being burnout predictors. Investigating the prevalence of burnout and distress in health-care staff in a postdisaster setting and identifying predictors of burnout development such as stress levels, time-management skills and work-life balance will contribute to the development of preventative strategies and better organization at work with a view to improving public health efficacy and reducing public health costs, given that these workers live in the disaster-affected community as survivors and serve as disaster relief workers at the same time.

## Introduction

Burnout is a work-related mental health impairment. There is still no universally accepted definition of this phenomenon: in fact, it still does not appear in the most recent versions of the most common classification systems (ICD-10 and DSM-5) (1, 2), but most researchers favor the multifaceted definition developed by Maslach and Jackson, which describes job burnout in terms of three components: Emotional Exhaustion (EE), Depersonalization (D), and the lack of Personal Accomplishment (PA) (3). EE is used to describe a state of feeling emotionally overextended and exhausted by work; DP refers to negative and cynical attitudes toward work; reduced PA denotes feelings of incompetence, inefficiency, and inadequacy.

However, morbidity associated with these conditions and their impact on the workplace is receiving increasing attention from society and the medical field (4).

Several studies indicate that health workers are the professionals with the highest prevalence of burnout, ranging from 30 to 70% (5) and with the greatest risk of long-term psychopathological consequences, such as anxiety, depression, substance abuse, and risk of suicide (5).

Burnout, in fact, is caused by the stress that results from the social relationship between a helper and a help recipient, usually found in asymmetrical professional relationships, whereby the victim is the “giver” and the client(s) the “receiver” (6).

Cataclysmic events such as earthquakes can be tremendous sources of stress (7–9), and studies analyzing the prevalence of burnout in a postnatural disaster setting showed different results. Two years after the 2011 Great East Japan Earthquake, Fujitani et al. found that a significant number of caregivers showed signs of EE, low PA, and psychological distress using the Maslach Burnout Inventory for Human Services (MBI-HS) and the General Health Questionnaire 12 Items (GHQ-12) as tools for measuring burnout and psychiatric symptoms, respectively (10). Instead, James et al. in the aftermath of the 2010 Haiti earthquake assessing a sample of mental health workers (the earthquake survivors themselves) demonstrated low burnout among the quantitative results and satisfaction associated with helping others among the qualitative results (11).

In the early morning of April 6, 2009, L’Aquila, capital city of the Abruzzo region and seat of the regional hospital, was struck by a 6.3 magnitude earthquake, followed by approximately 20,000 aftershocks. The earthquake caused significant damage to the thirteenth-century town of L’Aquila and to several medieval hill villages in nearby areas. Institutional sources reported 309 residents dead, over 1,600 people injured, and approximately 70,000 people made homeless (12). In this postdisaster setting, Valenti et al. studied the burnout impact on a sample of therapists working with young people with autism, confirming that burnout was induced by exceptional stressors related to the earthquake: they reported higher levels of EE and lower PA scores in the exposed group compared to the unexposed group (13).

To our knowledge, the literature on postdisaster settings in Italy is limited in its focus on mental health professionals rather than other health-care workers. Our study aims to evaluate the prevalence of burnout and psychopathological distress in different categories of health-care workers 6 years after the 2009 L’Aquila earthquake, focusing on work-related stress and predictors of burnout in order to prevent and reduce work-related burnout.

## Materials and Methods

The data collection of this observational cross-sectional study was conducted on a representative sample of health-care workers, i.e., physicians, nurses, and health-care assistants, working in different departments at L’Aquila St. Salvatore General Hospital from February to April 2015. To select a representative sample of the entire population, a specific proportional weight for each department was attributed on the basis of the number of afferent personnel. Numerous departments were extracted without repetition until the total number of health-care workers reached the numerosity (number) expected for the calculation of the sample’s dimension. A two-stage cluster sampling was carried out with probability proportional to size, where every single department as a cluster, or all of the afferent personnel (doctors, nurses, health-care assistants) in the department as a detection unit were considered. Thus, a total of 8 departments were selected, with probability proportional to size, from a total of 28 (for a total population of 535 health-care workers). The total sample included 300 health-care workers selected using simple random sampling. The research was conducted according to the requirements of Good Clinical Practice of the European Union and the current revision of the Helsinki Declaration.

The protocol was approved by the ethics committee (Local Health Authority ASL 1 Avezzano, Sulmona, L’Aquila) in November 2013 (n. 0116554/13). Written informed consent was obtained from each participant who had voluntarily agreed to participate. We protected the privacy and anonymity of the individuals involved.

### Assessment

All the participants completed the following self-reporting questionnaires.

#### Sociodemographic Data Form

An *ad hoc* schedule was developed to evaluate the sociodemographic and the work-related and anamnestic features of the study participants, as well as their direct exposure to the L’Aquila earthquake.

#### Maslach Burnout Inventory (MBI) (14)

The MBI is a 22-question self-evaluation questionnaire designed by Maslach and Jackson to measure the three dimensions of burnout measured by a separate subscale: EE, D, and the lack of PA. EE (nine items) assesses the feelings of being emotionally overwhelmed and exhausted by work; D (five items) measures a negative, cynical attitude toward one’s work; PA (eight items) assesses the negative evaluation of one’s competence and achievements at work. All the questions are rated on a Likert scale ranging from 0 = Never to 6 = Every Day. High EE (≥24) is considered to be an indicator of high burnout, as D scores equal to or higher than 9, whereas a PA score below 29 is considered to be the cutoff score for the aggravation of the pathology.

#### General Health Questionnaire-12 Items (15)

The GHQ-12 is a 12-item self-reporting instrument for the detection of mental disorders in the community and in non-psychiatric clinical settings. It measures aspects of anxiety, depression and social functioning. Although it does not yield a diagnosis, positive scores are indicative of psychological distress. The GHQ-12 asks respondents to report how they have been feeling over the past few weeks using a 4-point scale (“more than usual, as usual, less than usual, and much less than usual”). It is scored using a continuous response format (Likert scale: 0-1-2-3), resulting in a scale ranging from 0 to 36. A score of <15 indicates normal levels of stress, a score between 15 and 20 indicates the presence of stress and a score of >20 indicates severe psychological distress.

### Statistical Analysis

The characteristics of the study sample were analyzed using descriptive statistics. The Kruskal–Wallis test was used to compare the average MBI and GHQ-12 scores for different professional categories. When the resulting differences were statistically significant, *post hoc* tests were used for pairwise comparisons, and the Bonferroni correction was used to counteract the problem of multiple comparisons (*p* < 0.0167).

Using the MBI score, the sample was stratified into two groups: those with a high level of burnout (high burnout) as the sum both of the participants in burnout (B) and with a high risk of burnout, and those without a high level of burnout (a low or average risk of developing this condition) (16).

The discrete and nominal variables were expressed using frequencies and percentages, and the χ^2^ test or the χ^2^ test for the trend for ordinal variables were used to examine differences between the two groups. When the expected frequencies for the cells of the contingency table were fewer than 5, we used the Fisher’s exact test. Continuous variables were expressed as mean values and relative SDs, the differences of which in the absence/presence of high burnout were tested using the Wilcoxon–Mann–Whitney test, as the non-normal data distribution (Shapiro–Wilk test).

Univariate and multiple logistic regressions were performed to identify predictors of high burnout, with associations reported as odds ratios (ORs) and 95% confidence intervals (95% CIs). Predictors that had a *p*-value of less than 0.05 in univariate models were included in the multiple logistic regression models. Backward stepwise selection with the Akaike information criterion (AIC) was used to determine the factors that best predicted high burnout.

All the data were recorded electronically, and statistical analyses were carried out using the Stata Statistical Software: Release 12 (Stata Corp LP, College Station, TX, USA). All the tests were two tailed, and *p*-values ≤ 0.05 were considered statistically significant.

## Results

A total of 284 health professionals agreed to participate in the study, accounting for 94.67% of the total enrolled sample population (300 subjects).

According to the scores obtained from the MBI questionnaire (Table 1), we found a high level of EE (scores ≥ 24) in 36.97% of the respondents, a high level of D (≥9 scores) in 23.24% and a low PA (scores ≤ 29) in 21.83%. On the basis of these results, the sample was stratified into two groups: one group with a high level of burnout (23.24%) and another group without high burnout (76.76%). Through the administration of the GHQ-12 (Table 1), a high level of perceived stress among the respondents emerged, with stress and psychological distress for 32.04 and 20.42% of the respondents, respectively.

**Table 1:** Distribution of the sample for the three dimensions of burnout according to the Maslach Burnout Inventory and the prevalence of psychological distress.

MBI	*n* (%)	Mean ± SD
**Emotional exhaustion (EE)**	284	20.86 ± 14.55
Low (≤14)	117 (41.20)	7.30 ± 4.26
Average (15–23)	62 (21.83)	18.77 ± 2.51
High (≥24)	105 (36.97)	37.19 ± 8.58
**Depersonalization (D)**	284	5.60 ± 5.86
Low (≤3)	136 (47.89)	0.93 ± 1.14
Average (4–8)	82 (28.87)	6.16 ± 1.22
High (≥9)	66 (23.24)	14.53 ± 4.20
**Lack of Personal Accomplishment (PA)**	284	35.24 ± 8.80
Low (≥37)	147 (51.76)	41.90 ± 3.43
Average (30–36)	75 (26.41)	33.09 ± 2.10
High (≤29)	62 (21.83)	22.03 ± 5.98
**Burnout**	284	
Low risk	139 (48.94)	
Average risk	79 (27.82)	
High risk (HR)	39 (13.73)	
In burnout (B)	27 (9.51)	
**High level of burnout**		
No	218 (76.76)	
Yes (AR + B)	66 (23.24)	
GHQ-12		
**Normal level of stress**	135 (47.54)	10.79 ± 2.64
**Presence of stress**	91 (32.04)	17.27 ± 1.63
**Pathological distress with psychological aftermath**	58 (20.42)	25.48 ± 4.59

Among the professional categories (Table 2), statistically significant differences emerged solely for the total scores of GHQ-12: the *post hoc* analysis showed that the total average GHQ-12 scores were significantly higher in doctors than in health-care assistants (16.64 ± 6.29 vs. 13.65 ± 5.15, *p* = 0.010).

**Table 2:** Burnout dimensions and stress levels in the different professional categories.

	Professional categories				
	Total	Doctor (D)	Nurse (N)	Health-care assistant (HA)	*p*-Value*

	*N* = 284	*n* = 77	*n* = 173	*n* = 34	

	Average score ± SD	Average score ± SD	Average score ± SD	Average score ± SD	
**MBI**					
Emotional exhaustion (EE)	20.86 ± 14.55	20.75 ± 14.24	21.25 ± 14.86	19.06 ± 13.96	0.747
Depersonalization (D)	5.60 ± 5.86	6.47 ± 6.17	5.45 ± 5.73	4.41 ± 5.65	0.071
Lack of personal accomplishment (PA)	35.24 ± 8.80	35.73 ± 8.38	34.58 ± 8.91	37.50 ± 8.95	0.118
**GHQ-12**	15.87 ± 6.35	16.64 ± 6.29	15.96 ± 6.52	13.65 ± 5.15	**0.043**
Normal level of stress	10.79 ± 2.64	10.81 ± 2.83	10.78 ± 2.56	10.77 ± 2.74	0.377
Presence of stress	17.27 ± 1.63	17.62 ± 1.72	17.15 ± 1.61	16.89 ± 1.36	0.972
Pathological distress with psychological aftermath	25.48 ± 4.59	25.59 ± 3.87	25.47 ± 5.00	25.00 ± 4.36	0.920
***Post hoc* analysis**					
GHQ-12	*p*-Value**		*p*-Value**	*p*-Value**	
	D vs. N		D vs. HA	N vs. HA	
	n.s.		0.010	n.s.	

*Kruskal–Wallis test.

**Wilcoxon–Mann–Whitney test with the Bonferroni correction for multiple comparisons (*p* < 0.0167).

We compared the two groups (presence or absence of high burnout) for the sociodemographic and work-related characteristics, medical history, earthquake experience, social functioning, and stress levels.

Table 3 describes the sample’s sociodemographic characteristics. 56.69% were female, whose mean age was 43.44 ± 11.38 years. The most represented age group was 20–39 years, accounting for 37.82% of respondents. 66.20% were married and 60.21% had children. There were no statistically significant differences between the two groups and no association with high burnout for gender, age, marital status, or the presence of children was found.

**Table 3:** Sociodemographic variables of the sample according to the absence/presence of high burnout as percentages (%) or mean values with SDs and their association with high burnout by univariate logistic regression.

	High burnout				Univariate logistic regression		
	Total	No	Yes	*p*-Value	OR	95% CI	*p*-Value
	
	*N* = 284	*n* = 218	*n* = 66				
Gender				0.493*			
Male^a^	123 (43.31)	92 (42.20)	31 (46.97)		1		
Female	161 (56.69)	126 (57.80)	35 (53.03)		0.82	0.47–1.43	0.494
Age				0.571*			
20–39 years^a^	104 (37.82)	80 (37.74)	24 (38.10)		1		
40–50 years	74 (26.91)	60 (28.30)	14 (22.22)		0.78	0.37–1.63	0.505
>50 years	97 (35.27)	72 (33.96)	25 (39.68)		1.16	0.61–2.20	0.657
Mean ± SD	43.44 ± 11.38	43.41 ± 11.13	43.54 ± 12.26	0.970**	1.00	0.98–1.03	0.937
Marital status				0.424*			
Unmarried^a^	96 (33.80)	71 (32.57)	25 (37.88)		1		
Married	188 (66.20)	147 (67.43)	41 (62.12)		0.79	0.45–1.40	0.425
Presence of children				0.618*			
No^a^	113 (39.79)	85 (38.99)	28 (42.42)		1		
Yes	171 (60.21)	133 (61.01)	38 (57.58)		0.87	0.50–1.52	0.618

*^a^Reference category*.

*χ^2^ test or χ^2^ test for trend.

**Wilcoxon–Mann–Whitney test.

Table 4 shows aspects related to work experience. The majority of the subjects enrolled in the study were nurses (60.92%). The prevalence of high burnout was 25.97%, 20/77 in doctors and 22.54%, 39/173% in nurses. The average number of years of work in health care amounted to 16.95 ± 11.73, and the majority of the sample (62.32%) reported having worked in health care for more than 10 years. The average number of years in the same department was a little more than 10 years (10.85 ± 10.30), and 59.01% of the sample said they worked 36 h/week. The percentage of those who worked more than 5 overtime hours per week was 60.95%. Statistically significant differences (*p* < 0.001) between the two groups emerged for job perception and relationship with colleagues, with a prevalence of high job perception (71.21 vs. 36.87%) and a higher percentage of hostile relationships with colleagues (48.48% vs. 15.60) among the high burnout group compared to the other group. The hostile relationships with colleagues appeared to be a potential predictor of burnout (OR 5.09, 95% CI 2.78–9.33; *p* < 0,001).

**Table 4:** Work-related variables of the sample according to the absence/presence of high burnout as percentages (%) or mean values with SDs and their association with high burnout by univariate logistic regression.

	High burnout				Univariate logistic regression		
	Total	No	Yes	*p*-Value	OR	95% CI	*p*-Value
	
	*N* = 284	*n* = 218	*n* = 66				
Occupation				0.777*			
Doctor^a^	77 (27.11)	57 (26.15)	20 (30.30)		1		
Nurse	173 (60.92)	134 (61.47)	39 (59.09)		0.83	0.45–1.54	0.556
Health-care assistant	34 (11.97)	27 (12.39)	7 (10.61)		0.74	0.28–1.96	0.543
Time of work (years)				0.930*			
<5^a^	60 (21.13)	45 (20.64)	15 (22.73)		1		
5–10	47 (16.55)	36 (16.51)	11 (16.67)		0.92	0.38–2.24	0.849
>10	177 (62.32)	137 (62.84)	40 (60.61)		0.88	0.44–1.73	0.704
Mean ± SD	16.95 ± 11.73	16.92 ± 11.62	17.06 ± 12.19	0.943**	1.00	0.98–1.02	0.933
Time of work in actual ward (years)				0.653*			
<5^a^	113 (39.93)	90 (41.28)	23 (35.38)		1		
5–10	61 (21.55)	45 (20.64)	16 (24.62)		1.39	0.67–2.89	0.376
>10	109 (38.52)	83 (38.07)	26 (40.00)		1.23	0.65–2.31	0.530
Mean ± SD	10.85 ± 10.30	10.56 ± 10.12	11.82 ± 10.89	0.496**	1.01	0.99–1.04	0.388
Working hours per week				0.259*			
36^a^	167 (59.01)	132 (60.83)	35 (53.03)		1		
>36	116 (40.99)	85 (39.17)	31 (46.97)		1.38	0.79–2.40	0.260
Mean ± SD	39.18 ± 5.85	39.11 ± 6.05	39.41 ± 5.16	0.274**	1.01	0.96–1.06	0.712
Working overtime hours per week				0.488*			
<5^a^	50 (29.59)	32 (26.89)	18 (36.00)		1		
5	16 (9.47)	12 (10.08)	4 (8.00)		0.59	0.17–2.11	0.420
>5	103 (60.95)	75 (63.03)	28 (56.00)		0.66	0.32–1.37	0.266
Mean ± SD	6.22 ± 4.13	6.32 ± 4.17	5.96 ± 4.06	0.552**	0.98	0.90–1.06	0.593
Job perception				<0.001*			
Low^a^	17	15 (6.91)	2 (3.03)		1		
Moderate	139	122 (56.22)	17 (25.76)		1.05	0.22–4.97	0.956
High	127	80 (36.87)	47 (71.21)		4.41	0.96–20.12	0.056
Relationship with colleagues				<0.001*			
Friendly^a^	218 (76.76)	184 (84.40)	34 (51.52)		1		
Hostile	32 (48.48)	34 (15.60)	32 (48.48)		5.09	2.78–9.33	**<0.001**

*^a^Reference category*.

*χ^2^ test or χ^2^ test for trend.

**Wilcoxon–Mann–Whitney test.

As shown in Table 5, the percentage of those who were directly exposed to the L’Aquila earthquake was 43.66%, and 16.90% experienced grief; psychopathological history was reported by 59.57% of the participants, and requests for help and treatment involved 25.69 and 20.07% of the study sample, respectively. The lack of social functioning was more common in the high burnout group (13.64 vs. 2.30%), as well as the pathological distress with the psychological aftermath (56.06 vs. 9.63%).

**Table 5:** Anamnestic, related to the earthquake experience, social functioning variables, and the sample’s levels of stress according to the absence/presence of high burnout as percentages (%) or mean values with SDs and their association with high burnout by univariate logistic regression.

	High burnout				Univariate logistic regression		
	Total	No	Yes	*p*-Value*	OR	95% CI	*p*-Value
	
	*N* = 284	*n* = 218	*n* = 66				
Direct exposure to the L’Aquila earthquake				0.142			
No^a^	160 (56.34)	128 (58.72)	32 (48.48)		1		
Yes	124 (43.66)	90 (41.28)	34 (51.52)		3.05	1.70–5.46	**0.001**
Grief				0.069			
No^a^	236 (83.10)	186 (85.32)	50 (75.76)		1		
Yes	48 (16.90)	32 (14.68)	16 (24.24)		1.86	0.95–3.66	0.072
Psychological history				0.218			
No^a^	114 (40.43)	92 (42.40)	22 (33.85)		1		
Yes	168 (59.57)	125 (57.60)	43 (66.15)		1.44	0.81–2.57	0.219
Help request				0.497			
No^a^	56 (25.69)	45 (26.79)	11 (22.00)		1		
Yes	162 (74.31)	123 (73.21)	39 (78.00)		1.30	0.61–2.75	0.497
Treatment				0.931			
No^a^	227 (79.93)	174 (79.82)	53 (80.30)		1		
Yes	57 (20.07)	44 (20.18)	13 (19.70)		0.97	0.49–1.94	0.931
Social functioning				**0.001**			
Good^a^	40 (14.13)	32 (14.75)	8 (12.12)		1		
Moderate	229 (80.92)	180 (82.95)	49 (74.24)		1.09	0.47–2.51	0.842
Nothing	14 (4.95)	5 (2.30)	9 (13.64)		7.20	1.89–27.49	**0.004**
GHQ-12				**<0.001**			
Normal level of stress^a^	135 (47.54)	124 (56.88)	11 (16.67)		1		
Presence of stress	91 (32.04)	73 (33.49)	18 (27.27)		2.78	1.24–6.21	**0.013**
Pathological distress with psychological aftermath	58 (20.42)	21 (9.63)	37 (56.06)		19.86	8.78–44.95	**<0.001**

*^a^Reference category*.

*χ^2^ test or χ^2^ test for trend.

Direct exposure to the L’Aquila earthquake (OR 3.05, 95% CI 1.70–5.46; *p* < 0.001), lack of social functioning (OR 7.20, 95% CI 1.89–27.49; *p* = 0.004) and moderate (OR 2.78, 95% CI 1.24–6.21; *p* = 0.013) to high levels of stress (OR 19.86, 95% CI 8.78–44.95; *p* < 0.001), other than hostile relationships with colleagues, resulted in being potential predictors of burnout. Only three of these four potential predictors were included in the multivariate analysis (Table 6), according to the stepwise selection based on AIC. Hostile relationships with colleagues (OR 3.34, 95% CI 1.63–6.483; *p* = 0.001), direct exposure to the L’Aquila earthquake (OR 2.52, 95% CI 1.28–4.94; *p* = 0.007) and moderate (OR 2.61, 95% CI 1.14–5.96; *p* = 0.023) to high levels of distress (OR 14.38, 95% CI 6.10–33.88; *p* < 0.001) emerged as significant predictors of high burnout.

**Table 6:** Multivariate logistic regression analysis of risk factors associated with burnout.

		High burnout		Multivariate logistic regression		
		
	Total	No	Yes	OR^b^	95% CI	*p*-Value
	
	*N* = 284 (%)	*n* = 218 (%)	*n* = 66 (%)			
**Relationship with colleagues**						
Friendly^a^	218 (76.76)	184 (84.40)	34 (51.52)	1		
Hostile	66 (23.24)	34 (15.60)	32 (48.48)	3.34	1.63–6.83	**0.001**
**Direct exposure to the L’Aquila earthquake**						
No^a^	160 (56.34)	128 (58.72)	32 (48.48)	1		
Yes	124 (43.66)	90 (41.28)	34 (51.52)	2.52	1.28–4.94	**0.007**
**GHQ-12**						
Normal level of stress^a^	135 (47.54)	124 (56.88)	11 (16.67)	1		
Presence of stress	91 (32.04)	73 (33.49)	18 (27.27)	2.61	1.14–5.96	**0.023**
Pathological distress with psychological aftermath	58 (20.42)	21 (9.63)	37 (56.06)	14.27	6.10–33.88	**<0.001**

*^a^Reference category*.

*^b^Adjusted odds ratios for the other variables in the model*.

## Discussion

Our cross-sectional investigation analyzed burnout and distress levels among caregivers working at St. Salvatore General Hospital, 6 years after the 2009 L’Aquila earthquake. The study setting was characterized at the same time by prolonged exposure to one of the most stressful work contexts in the postearthquake aftermath. The results demonstrated that direct exposure to the earthquake was a significant predictor of high burnout. In fact, local workers not only experienced the disaster themselves, but were also under pressure to respond to others’ needs in the aftermath of the earthquake as well as to completing their regular duties (17).

This finding highlights the need to implement long-term psychological support, particularly with regard to those who survived natural disasters to help them to process the trauma and to improve their stress coping strategies, as their psychological well-being has an important influence on the quality of care available for patients (9, 10, 18).

There is a lack of research into the effects on caregivers’ mental health after a disaster in the Italian context, but some studies are available in the same setting we considered in this investigation: Valenti et al. demonstrated that therapists of children and adolescents with autism in the group exposed to the earthquake appeared to report significantly higher levels of EE or lower levels of PA than the non-exposed staff (13). Although we did not consider any non-exposed group, the EE levels in our study group’s results were in line with those of the exposed group, as well as with those in a previous study on hospital professionals; being victims themselves they may have developed a conflict of roles and assumed too much responsibility at work (19).

This observation might also justify the high observed GHQ-12 total score. In particular, doctors showed a significantly higher score than health-care assistants. In contrast to previous findings by Guveli et al. (20), which revealed higher EE levels among doctors and nurses compared to other professionals, our study showed no statistically significant differences in burnout levels between the professional categories.

The prevalence of high burnout among doctors (25.97%, 20/77) was in line with the results reported for physicians in the USA (22%), in Great Britain (27%), and in Germany (20%) (6), in a review by Awa et al. (6). Moreover, in contrast with a previous study, nursing personnel did not emerge as being more susceptible to burnout (22.54%, 39/173) compared to other health-care workers (21). However, the prevalence of burnout in our study should not be compared directly, as different definitions of burnout were considered in the cited studies.

In line with previous studies, other factors emerged as potential predictors of burnout development, such as a high perception of workload and hostile relationships with colleagues (22–26), as they were predisposed to feelings of exclusion and isolation; Leon-Perez et al. demonstrated that preventing intragroup conflict could improve employees’ well-being and performance, thus decreasing burnout and increasing quality of service (27).

Good relations between colleagues, which seems to have a protective role against burnout (28), should be encouraged to improve teamwork and collaboration. The role of hostile relationships with colleagues as a significant predictor of high burnout was confirmed by our multiple logistic regression.

In health-care staff other already known potential predictors of burnout after exposure to a natural disaster were low social support and work-related stress, such as high work loads and long working hours (29).

Work-related stress emerged as another significant predictor of high burnout by our multivariate regression analysis. As burnout is defined as a psychological response to work-related stress (14) in health-care settings, health workers are continuously exposed to a variety of difficult to manage stressors, including the burden of responsibility and excessive work, the management of complex situations, excessive demands from patients, the uncertainty of their treatments, painful medical procedures, potential death scenarios, the absence of social support, and not always being able to develop adaptive emotional reactions (7). Therefore, it would be important to periodically monitor the mental health status of health workers and improve preventative strategies so as better to recognize the first signs of stress thus reducing the prevalence of burnout (25, 29).

By using a univariate logistic model, a lack of social functioning was also found to be a potential predictor of high burnout. In fact, one of the predisposing behavioral characteristics to this condition is to consider work as a surrogate for social life (30). Therefore, preventative interventions might also include an improvement in time management and in the balance between private and professional lives (31). Nevertheless, the role of this factor as being significant in predicting burnout was not confirmed by our multiple regression analysis.

There are several limitations in this study. We conducted a cross-sectional study; therefore, we cannot define causal relationships between sociodemographic data, burnout and psychological distress. These findings should be confirmed in a future longitudinal study, which would more clearly delineate the role of these variables on burnout and psychological distress. Moreover, all the data were based on self-reported questionnaires, and the potential problem of variance with this common method cannot be avoided (32). In addition, there is more than one burnout classification, but in this study, we considered only the three main classically recognized sub-psychopathological dimensions. Finally, as our interest was on burnout and work-related stress, posttraumatic stress disease assessment was not considered relevant especially as in the interview period of 6 years other factors could have affected the outcome and the study was focusing specifically on the workplace.

In conclusion, investigating the prevalence of burnout and distress in health-care staff in a postdisaster setting appears to be relevant when directing attention toward issues related to stress, to time management at work, to supervision, and to the balance between private and professional lives. This will improve organization at work, thus improving public health efficacy and reducing public health costs, as these workers live in the disaster-affected community as survivors and serve as disaster relief workers at the same time.

## Ethics Statement

The Ethical approval was obtained in November, 2013 from the ethical review committee of the Local Health Unit—n. 0116554/13 (ASL 01—Avezzano, Sulmona, L’Aquila).

## Author Contributions

AM conceived the study, participated in its design and in drafting the manuscript, FF participated in the study design and was involved in drafting the manuscript, MM managed the questionnaires. AM performed the statistical analysis, VB has made substantial contribution to the study conception and revised the manuscript. SN collaborated to the revised article. All authors read and approved the final version of the manuscript.

## Conflict of Interest Statement

The authors declare that the research was conducted in the absence of any commercial or financial relationships that could be construed as a potential conflict of interest. The reviewer, DB-B, and handling editor declared their shared affiliation, and the handling editor states that the process nevertheless met the standards of a fair and objective review.
